# A Reversibly Thermoresponsive, Theranostic Nanoemulgel for Tacrolimus Delivery to Activated Macrophages: Formulation and In Vitro Validation

**DOI:** 10.3390/pharmaceutics15102372

**Published:** 2023-09-22

**Authors:** Riddhi Vichare, Caitlin Crelli, Lu Liu, Amit Chandra Das, Rebecca McCallin, Fatih Zor, Yalcin Kulahci, Vijay S. Gorantla, Jelena M. Janjic

**Affiliations:** 1 School of Pharmacy, Graduate School of Pharmaceutical Sciences, Duquesne University, Pittsburgh, PA 15282, USA; vicharer@duq.edu (R.V.); crellic@duq.edu (C.C.); liul@duq.edu (L.L.); dasa@duq.edu (A.C.D.); mccallinr@duq.edu (R.M.); 2 Wake Forest School of Medicine, Wake Forest Institute of Regenerative Medicine, Winston Salem, NC 27101, USA; fzor@wakehealth.edu (F.Z.); ykulahci@wakehealth.edu (Y.K.); vgorantl@wakehealth.edu (V.S.G.)

**Keywords:** nanoemulgel, tacrolimus, transplantation, nanoemulsion, macrophages

## Abstract

Despite long-term immunosuppression, organ transplant recipients face the risk of immune rejection and graft loss. Tacrolimus (TAC, FK506, Prograf^®^) is an FDA-approved keystone immunosuppressant for preventing transplant rejection. However, it undergoes extensive first-pass metabolism and has a narrow therapeutic window, which leads to erratic bioavailability and toxicity. Local delivery of TAC directly into the graft, instead of systemic delivery, can improve safety, efficacy, and tolerability. Macrophages have emerged as promising therapeutic targets as their increased levels correlate with an increased risk of organ rejection and a poor prognosis post-transplantation. Here, we present a locally injectable drug delivery platform for macrophages, where TAC is incorporated into a colloidally stable nanoemulsion and then formulated as a reversibly thermoresponsive, pluronic-based nanoemulgel (NEG). This novel formulation is designed to undergo a sol-to-gel transition at physiological temperature to sustain TAC release in situ at the site of local application. We also show that TAC-NEG mitigates the release of proinflammatory cytokines and nitric oxide from lipopolysaccharide (LPS)-activated macrophages. To the best of our knowledge, this is the first TAC-loaded nanoemulgel with demonstrated anti-inflammatory effects on macrophages in vitro.

## 1. Introduction

The World Health Organization (WHO) estimates that the current organ transplantation rate meets only about 10% of the global transplantation need [1], as the demand has grown over sixfold in the last three decades [2]. As of March 2023, in the United States alone, 104,234 patients were waiting for a transplant, with one patient being added to the list every hour. Sadly, nearly half of the 42,000 organ transplants that were performed in 2022 [3] will be rejected within the first 10 years by the recipients, despite being under broad immunosuppression [4]. This is because, even though our understanding of the cellular and molecular immune mechanisms that lead to rejection has exponentially grown, the non-specific approach of using high-dose, multi-drug, systemic immunosuppressive medications has remained the standard of care in transplantation for over two decades [2,5].

Recent studies in organ transplants have suggested that host monocyte-derived macrophages drive acute rejection (AR) and chronic rejection (CR) by upregulating activated (effector) T cells [6]. Early macrophage infiltration has been correlated with a poor prognosis after transplantation. Importantly, these graft-infiltrated macrophages were skewed towards the proinflammatory phenotype (referred to as M1), consequently increasing the risk of rejection [7]. Importantly, the intensity of graft-infiltrating macrophages has been shown to reliably correlate with the risk of graft rejection [8], and the depletion of macrophages in the graft has been found to inhibit both AR and CR [9]. Macrophages contribute to transplant rejection through various pathways, such as releasing pro-inflammatory cytokines (e.g., TNF-α, IL-β, IL-6), producing nitric oxide (NO), expressing cell surface costimulatory markers, and antigen presentation leading to the activation of recipient T cells in the transplant [10,11]. Macrophage cytokines such as IL-6 induce Th17 cell differentiation, which is positively associated with the degree of acute organ rejection [12,13].

Tacrolimus (TAC, FK 506, Prograf^®^) is an FDA-approved macrolide immunosuppressant drug isolated from the bacterium *Streptomyces tsukubaensis* [14]. TAC is 100 times more potent than cyclosporine (CSA) in reducing AR and improving graft survival [15]. However, TAC is a hydrophobic drug with a narrow therapeutic index and undergoes extensive first-pass metabolism with variable absorption, which results in fluctuating diurnal blood levels. The oral bioavailability of TAC ranges between 7.3% and 19.7% [16], and around 95% of the bioavailable TAC binds to the red blood cells (RBCs) after entering systemic circulation, where it exerts no immunomodulatory effects [16,17]. Consequently, this necessitates repeated administration of high systemic doses of TAC, which can result in adverse events such as chronic nephrotoxicity, diabetes mellitus, hypercholesterolemia, and hypertension [18]. The immunosuppressive mechanism of TAC primarily involves the inhibition of the calcineurin pathway and mitogen-activated protein kinase (MAPK) signaling pathways in T cells [19]. However, calcineurin and MAPK pathways are also present in macrophages [20,21]. Thus, the immunomodulatory effects of TAC on the monocyte-macrophage lineage are increasingly being recognized [22,23,24]. For instance, Kannegieter et al. [23] showed that the expression of cell surface markers for the M2/anti-inflammatory phenotype (CD200R and CD16) significantly increased in the presence of a high concentration of TAC (200 ng/mL). In LPS-activated human monocytic THP-1 cells, which differentiate into macrophages, TAC inhibited extracellular signal-regulated kinase (ERK), a member of the MAPK pathway, and decreased the release of tumor necrosis factor-α (TNF-α) [24].

Another major cause of delayed AR or CR that can lead to premature graft loss after successful transplantation is non-adherence to immunosuppressive medications [25,26]. Local delivery of low-dose immunosuppressive drugs has demonstrated superior outcomes to prevent organ rejection while avoiding global systemic exposures [27,28,29,30]. The use of long-acting controlled or sustained-release injectables or implants for immunosuppressive drug delivery directly to the graft has shown promise in transplants and has the potential to reduce the risk of non-adherence with oral medications [31]. However, none of the current platforms allow direct targeting or intracellular drug delivery to immune cells. Here, we propose a locally injectable, sustained release, graft- and immune cell-targeted delivery of TAC, which could either eliminate or reduce the need for the frequent administration of immunosuppressive drugs. This work thus focuses on developing an adjunctive or complementary site-specific drug delivery strategy with the prospect of improving overall compliance with oral or systemic immunosuppressive agents. In this work, we first formulated a colloidally and fluorescently stable near-infrared fluorescence (NIRF)-labeled, ultra-low-dose, TAC-containing nanoemulsion (NE). We then incorporated the NIRF-labeled TAC-NE in a nanoemulgel (NEG) platform built from Pluronics^®^, which are amphiphilic triblock copolymers consisting of poly-propylene oxide (PPO) and poly-ethylene oxide (PEO) units. NEGs are nanoemulsion-incorporated gels that have the potential to serve as sustained drug delivery systems upon application [32].

**Figure 1:**
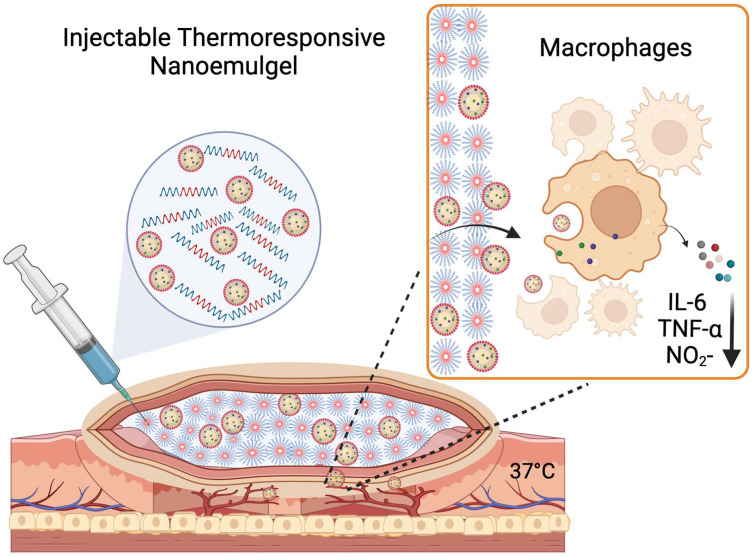
Illustration of proposed application of tacrolimus nanoemulgel (Created with Biorender.com).

In the presented study, we prepared a reversibly thermo-responsive NEG built from Pluronic F-127 (PEOxPPOyPEOx molecular ratio of 99:67:99) and Pluronic F-68 (PEOxPPOyPEOx molecular ratio of 76:30:76) that undergoes sol-to-gel transitions from ambient to physiological temperature (37 °C). Pluronics^®^ are reported to demonstrate “thermoreversible gelation” due to the temperature-dependent changes in the viscosity. The temperature-dependent solubility of PPO and PEO units in water promotes self-assembly and micellization [33]. The hydrophobic PPO segments aggregate to form the core of micelles, while the hydrated PEO segments form the outer shell. As the temperature increases, these micelles organize to give the system a gel-like consistency [34,35]. Both the Pluronics^®^ used in this work are US FDA-approved for drug delivery applications [33,36]. This work introduces, for the first time, to the best of our knowledge, the concept of an injectable thermoresponsive and theranostic NEG that can incorporate two payloads (TAC and near-infrared fluorescent dye) and demonstrates the in vitro uptake, safety, and therapeutic effectiveness of TAC-NEG to suppress TNF-α, IL-6, and NO production in LPS-activated macrophages. We also report on the colloidal and fluorescence properties, rheological measurements, sustained release, macrophage intracellular uptake of TAC from NEG, and long-term stability studies. A schematic illustration of the proposed application of TAC-NEG (Figure 1).

## 2. Materials and Methods

### 2.1. Materials

Tacrolimus (TAC, FK-506) was purchased from TSZCHEM, Framingham, MA, USA. Miglyol 812 was purchased from CREMER Oleo Product Division, Hamburg, Germany. The solubilizer, 2-(2-Ethoxyethoxy) ethanol (Transcutol) was obtained from Spectrum, New Brunswick, NJ, USA. The non-ionic surfactant, Pluronic^®^ P105 was procured from United States Biological, Salem, MA, USA, and Kolliphor^®^ EL (CrEL) was purchased from Sigma, St. Louis, MO, USA. Near-infrared fluorescent (NIRF) dye-(1,1′-Dioctadecyl-3,3,3′,3′-Tetramethylindodicarbocyanine, 4-Chlorobenzenesulfonate Salt) (DiD, 644 nm/663 nm) was purchased from Thermo Fischer Scientific, Waltham, MA, USA. The CellTiter-Glo^®^ 2.0 Luminescent Cell Viability Assay kit was purchased from Promega Corporation, Fitchburg, WI, USA. Human serum (H4522) was gifted by Millipore Sigma (St. Louis, MO, USA). Pluronic F-127 (Poloxamer 407) was purchased from Sigma-Aldrich (St. Louis, MO, USA) and Pluronic F-68 (Poloxamer 188) was purchased from Spectrum Chemical Mfg. Corp. (New Brunswick, NJ, USA). The enzyme-linked immunosorbent assay (ELISA) kit for mouse TNF-α was purchased from R&D Biosystems (catalog no. DY410-05), Minneapolis, MN, USA, and mouse IL-6 (catalog no. 88-70-64-88) was purchased from Invitrogen, Waltham, MA, USA. An adherent mouse macrophage cell line (RAW 264.7) was purchased from the American Type Culture Collection (ATCC TIB-71), Rockville, MD, USA, and cultured following the manufacturer’s instructions. For cell culture experiments, Dulbecco’s Modified Eagle Medium (DMEM; Gibco-BRL, Rockville, MD, USA) was supplemented with 10% fetal bovine serum (FBS, ATCC 3020-20). DMEM without Phenol Red (1X, ref. A14430-01) was obtained from Gibco, Thermo Fischer Scientific, Waltham, MA, USA. The Nitrite/Nitrate Assay Kit was purchased from Millipore Sigma (catalog no. 23479), St. Louis, MO, USA.

### 2.2. Methods

#### 2.2.1. Preparation of Nanoemulsions (NEs)

Nanoemulsions were manufactured using high-pressure homogenization following a previously reported procedure [37]. Briefly, prior to manufacturing, the interaction chamber of the M110S microfluidizer (Microfluidics Corporation, Westwood, MA, USA) was iced for 30 min. TAC (50 mg) was dissolved in 12.96% *w*/*v* of Miglyol 812 (hydrocarbon phase) along with 1.6% *w*/*v* of Transcutol (co-solubilizer) by stirring overnight. A pre-emulsion was made by mixing solubilized TAC with DiD (NIRF) dye and 0.22 µM filtered micelle solution in PBS. The final concentration of the micelle solution in PBS was 5% *w*/*v*, where 2% *w*/*v* was P105 and 3% *w*/*v* was CrEL. The final DiD dye concentration was 50 µM. The pre-emulsion mixture was vortexed at high speed for ~10 s before pouring into the M110S inlet reservoir and processed for 30 pulses (6 passes) at an inlet air pressure of ~80 psi and an operating liquid pressure of ~15,000 psi. After 30 pulses, the final nanoemulsion product was released from the outshoot, and the final volume (27 mL) was noted. The same procedure was followed to manufacture drug-free nanoemulsion (DF-NE) to serve as a control. TAC-NE and DF-NE were each reproduced three times.

#### 2.2.2. Preparation of Nanoemulgels (NEGs)

For sterile NEG preparation, sterilize the planetary centrifugal mixer (THINKY Mixer), the cupholder, and the THINKY cup with 70% IPA. Pluronic F-127 was dissolved in sterile 1X PBS before autoclaving, and Pluronic F-68 was dissolved in HPLC-grade water before passing through a 0.22 μm cellulose filter. The physicochemical properties of F-127 are not significantly affected by autoclaving procedures [38]. The final concentration of F-127 and F-68 in NEG was 18% *w*/*v* and 1% *w*/*v*, respectively. The 0.22 µM sterile-filtered NEs (5 mL) along with F-127 and F-68 were weighed in a THINKY cup. For the control gel, 5 mL of 1XPBS instead of NE was added to the F-127 and F-68 blends. Throughout the process, the polymer solutions, mechanical pipettes, THINKY cup, and cupholder were kept cold on ice. The THINKY cup was placed in a cupholder, and a counterbalance dial inside the mixer was set to balance the weight. Mixing was carried out for 90 s at 2000 rpm. The formed NEG was poured into a sterile centrifuge tube. Drug-free nanoemulgel (DF-NEG) was formulated using DF-NE and following the same procedure. TAC-NEG and DF-NEG were each reproduced three times.

#### 2.2.3. Colloidal Characterization of the NEs and NEGs

Dynamic light scattering (DLS) was used to measure size distribution (described by a z-average droplet diameter (nm) and polydispersity index (PDI)) and zeta potential (mV) on a Zetasizer Nano (Malvern Instruments, Worcestershire, UK). All the formulated products were diluted 1:40 *v*/*v* in deionized water prior to any characterization by DLS to avoid multiple scattering effects. DLS operating parameters were set as follows: refractive indices of material, 1.59; refractive index of dispersant, 1.33; temperature, 25 °C; viscosity of the dispersant, 0.8872 cP; and backscatter angle, 173 degrees. DLS data is plotted as the mean ± standard deviation of three measurements.

#### 2.2.4. Physical Stress Testing of NEs: Centrifugation and Filtration

For the centrifugation test, NEs were centrifuged at 3000 rpm for 30 min at ambient temperature (Labnet Prism R Refrigerated Micro-Centrifuge). For the filtration test, NEs were filtered through a 0.22 µm pore mixed cellulose ester membrane (Merck Millipore Ltd., Burlington, MA, USA). Post physical stress, NEs were diluted 1:40 *v*/*v* in deionized water and characterized for droplet diameter and PDI.

#### 2.2.5. Thermal Cycling and Freeze-Thaw Cycling of NEs

The developed NEs were aliquoted into a 5 mL glass vial and sealed with parafilm. Four alternating cycles were performed between an oven temperature of 50 °C and a refrigerator temperature of 4 °C to evaluate the colloidal stability. For freeze-thaw cycles, NEs were aliquoted into a 5 mL glass vial and sealed with parafilm. The NEs were alternated between −20 °C and 25 °C for four cycles. NEs were diluted 1:40 *v*/*v* in deionized water and characterized for droplet diameter, PDI, and TAC loading before and after the completion of the cycles. The percentage change in droplet diameter after stress tests was recorded and reported from three measurements.

#### 2.2.6. Serum Stability Testing of NEs and NEGs

Serum stability was assessed in NEs as well as NEGs. For serum stability, NEs and NEGs were diluted 1:40 *v*/*v* in 10% human serum in colorless Dulbecco’s Modified Eagle’s Medium (DMEM). The diluted NEs were incubated at 37 °C for 72 h. DLS measurements (droplet diameter and PDI) were obtained at the beginning and end of 72 h of incubation.

#### 2.2.7. Near-Infrared Fluorescence (NIRF) Imaging of NEs and NEGs

NIRF imaging of NEs and NEGs was performed on the Li-COR Odyssey. Serial dilutions of nanoemulsion in deionized water were prepared such that the DiD concentration is in a range of 5 µM to 0.156 µM. Dilutions were transferred to a clear 96-well plate in triplicate and the fluorescence intensity was measured on the Li-COR Odyssey M imaging system. The wavelength was set to 700 nm for DiD channel, and imaging parameters such as intensity and focus were kept constant between analyses.

#### 2.2.8. Sterility Testing of NEGs

The method was adopted from USP 34 microbiological tests/71 sterility tests as previously described [39,40]. NEG was diluted at a 1:40 *v*/*v* ratio in (1) fluid thioglycolate medium (Millipore, Catalog STBMCTM12) and (2) trypcase soy broth (Millipore, Catalog STBMTSB12). The pH was immediately measured post-dilution and a snapshot of the appearance of the medium was taken. Fluid thioglycolate medium dilutions were stored at 35 °C and Trypcase soy broth dilutions were incubated at 25 °C for 14 days. After 14 days of incubation, pH was recorded, and a visual test was performed. Increased cloudiness or changes in pH indicated microorganism growth. For each test, medium or broth incubated without NEG was used as a negative control.

#### 2.2.9. pH Measurements of NEs and NEGs

The pH of NE and NEG was measured without any dilution using a calibrated PH700 Benchtop pH meter from Apera Instruments. All the pH measurements were performed in triplicate.

#### 2.2.10. Reverse Phase High-Performance Liquid Chromatography (RP-HPLC)

TAC loading in NE and NEG was measured using a validated RP-HPLC method on a Dionex Ultimate 3000. The method was based on isocratic elution of TAC using acetonitrile:water:orthophosphoric acid (75:25:0.05) as the mobile phase on a C18 column (Phenomex column) with UV detection at 250 nm. The retention time for TAC was 5.7 min when the flow rate was maintained at 0.75 mL/min and the column temperature was 60 °C. For TAC content analysis, NEs or NEGs were first diluted in pure acetonitrile. Further, acetonitrile supernatants were then diluted with deionized water to match the mobile phase. All drug content analyses were performed in triplicate. The HPLC standard curve for TAC with the limit of detection (LOD) and limit of quantification (LOQ) and a representative HPLC chromatograph showing the TAC peak are shown in Supplementary Figure S2A,B.

#### 2.2.11. Dissolution Test to Study the Release of TAC-NE from TAC-NEG

TAC-NEG (3 mL) was aliquoted into a sterile 50 mL falcon tube, capped and sealed with parafilm before transfer to a humidified 37 °C oven for ≥5 min (until complete gel formation). Warm 10% FBS in DMEM without Phenol-red medium was gently added into the falcon tube. The tube was tightly capped and covered with foil before being placed back into the 37 °C oven. At pre-defined intervals (0, 1, 3, 6, 24, 30, 48, 54, 72 h) 100 µL of medium was removed without disturbing the NEG and transferred to a 96-well plate for fluorescent study. The removed medium was replaced with 100 µL of fresh 10% FBS in colorless DMEM. NIRF signal intensity was measured on the Li-COR Odyssey M in a 700-nm channel at a fixed intensity and focus.

#### 2.2.12. In Vitro Release of TAC from TAC-NE and TAC-NEG

The quantitative in vitro release test was performed at 37 ± 0.5 °C using the dialysis bag technique (molecular cutoff of 3000 Dalton). A total of 1 mL of TAC NEG, an equivalent drug containing TAC NE, and TAC solution were placed in the snakeskin dialysis bag (ThermoFischer Scientific, REF 68035, USA). The dialysis bag containing TAC-NEG was kept at 37 ± 0.5 °C until it gelled and equilibrated. The receptor compartment consisted of a 15 mL mixture of 0.1% Tween 80 in phosphate-buffered saline (PBS; pH 7.4). As TAC is a hydrophobic drug, to maintain sink conditions, the dialysis bag was switched into fresh medium in a 50 mL centrifuge tube at regular intervals (6, 24, 48, 72, 168, 366, and 528 h). The quantitative analysis of TAC was performed using the HPLC method, as described in the “RP-HPLC analysis” section. The cumulative percentage of drug release versus time was plotted to evaluate the drug release pattern from a TAC NEG, TAC NE, and TAC solution.

#### 2.2.13. Rheology on NEGs

All rheology experiments were performed according to our optimized protocols. Oscillation amplitude sweep (OA), oscillation frequency sweep (OF), and temperature ramp studies were performed on control gel, DF-NEG, and TAC-loaded NEG. All the experiments were performed using the Discovery HR20 Rheometer (TA Instruments, Waters™, Milford, MA, USA). A 20 mm parallel plate stainless steel geometry was used with a 45 mm loading gap and a 1 mm geometry gap. The samples were kept on ice for at least 30 min before starting the rheology experiment and 0.36 mL was run for each sample. To ensure consistency, the samples were kept in ice during the total run time of the experiment, and the Peltier plate was brought down to 20–22 °C before the initiation of each run. The oscillation temperature ramp studies were performed from 20 to 50 °C with a ramp rate of 5 °C per minute. The oscillation strain was constant at 0.1% with a constant angular frequency of 10 rad/s. The amplitude sweep measurements were carried out after a 15-min wait time at 37 °C. The oscillation strain was varied from 0.1 to 100% with a constant angular frequency of 10 rad/s to determine the storage modulus (G’) and loss modulus (G”) as a function of oscillation strain%. Frequency sweep measurements were carried out using a constant oscillation strain of 0.1% and changing the angular frequency from 0.1 to 100 rad/s. This experiment was performed at 37 °C with 1 min of wait time. Temperature ramp (T-ramp) studies were performed from 5 to 50 °C and the changes in viscosity with increasing temperature were measured. T-ramp studies used a ramp rate of 5 °C per minute, a shear rate of 100/s, and a 60 s wait time. All the tests were performed on freshly prepared samples. Flow sweep experiments were performed at ambient temperature with increasing shear from 0.1 to 100 per second.

#### 2.2.14. Macrophage Viability following Exposure of NEs and NEGs

Raw 264.7 macrophages (passage numbers between 8 and 15) were seeded at 5000 cells per well in a 96-well plate and incubated overnight at 37 °C and 5% CO_2_. If macrophages were activated with LPS, the cell number was reduced to 3000 cells per well. Macrophages were activated using lipopolysaccharide (LPS, 500 ng/mL) for 18 h, followed by NE treatment for 24 h. According to the manufacturer’s instructions, the CellTiter Glo^®^ 2.0 A Luminescent Cell Viability Assay Kit (Promega) was used to assess cell viability by quantifying ATP in metabolically active and viable cells. Using untreated macrophages as a control, the percentage of viable macrophages was calculated using Equation (1).

(1) $$\%Cytotoxicity=\frac{\left[A\right]control-\left[A\right]test}{\left[A\right]contol}*100$$

where [A]test is the absorbance of the test sample and [A]control is the absorbance of the control sample.

#### 2.2.15. Fluorescence Microscopy

Raw 264.7 cells (passage numbers between 8 and 10) were seeded at 20,000 cells per well in an 8-well chamber slide (Lab-TekII) and left for attachment overnight. After 24 h of incubation at 37 °C and 5% CO_2_, the existing media was removed, and the cells were treated with either 20 μL/mL of NEs or 100 μL/mL of NEGs for 6 h. Then cells were thoroughly washed with warm 1X PBS before adding mounting media with DAPI at ambient temperature for at least 20 min prior to imaging on the Keyence BZX microscope.

#### 2.2.16. Flow Cytometry

For NE and NEG uptake, Raw 264.7 macrophages (passage number between 8 and 10) were plated in 12-well plates (0.2 million cells per well) and left for attachment overnight in a full cell culture medium. After aspiration of the existing media, activated macrophages were treated with TAC NE and drug concentration matched TAC NEG for 30 min, 60 min, and 180 min. Cells were collected by trypsinization and fixed at room temperature with 2% PFA in 1X PBS for 20 min. All experiments were performed in triplicate, and samples were analyzed using Attune Nxt recording 40,000 events. The nanoemulsion was detected in the RL1 (DiD, 644 nm/663 nm) channel. Gating was applied based on forward scatter (FSC) and side scatter (SSC), as shown in Supplementary Figure S6.

#### 2.2.17. Enzyme-Linked Immunosorbent Assay (ELISA)

Raw 264.7 macrophages (passage numbers between 8 and 10) were plated into 6-well plates (0.3 million cells per well) and incubated for 48 h at 37 °C and 5% CO_2_. The existing medium was replaced, and then for 24 h cells were treated with either TAC-NE (50 μM, 40 μM, 20 μM, and 5 μM), DF-NE (volume-matched to the highest concentration of TAC-NE), TAC-NEG (50 μM, 40 μM, 20 μM, and 5 μM), DF-NEG (volume-matched to the highest concentration of TAC-NEG). Post 24 h of incubation at 37 °C and 5% CO_2_ with the treatments, the existing medium was replaced with LPS (500 ng/mL) for 18 h. The supernatants were collected and spun at 4 °C and 1100 rpm for 5 min. Spun supernatants were stored at −80 °C for ELISA analysis. IL-6 ELISA (Invitrogen) and TNF-α (R&D Biosystems) plates were developed following the manufacturer’s protocol. Fluorescence measurements were obtained using a Synergy HTX plate reader (BioTek, Winooski, VT, USA).

#### 2.2.18. Nitric Oxide Assay

The levels of NO in the presence or absence of NE and NEG were measured based on measuring the levels of nitrite (NO_2_^_^) using a colorimetric assay. Briefly, 1500 cells per well were seeded into 96-well plates. Post 24 h of incubation at 37 °C and 5% CO_2_, cells were treated with either TAC-NE and DF-NE or TAC-NEG and DF-NEG for 24 h. The next day medium was replaced with an LPS-containing medium (200 ng/mL) for 18 h. The Nitrite/Nitrate colorimetric assay was performed according to the manufacturer’s protocol (Millipore Sigma).

#### 2.2.19. Statistical Analysis

The data obtained were expressed in terms of mean ± standard deviation (SD) values. The data were analyzed by unpaired two-tailed Student’s *t*-test. The statistical significance level was set at *p* < 0.05, and all the data were analyzed using GraphPad Prism v9.3.1.

## 3. Results

### 3.1. Preparation and Characterization of TAC-NE

**Figure 2:**
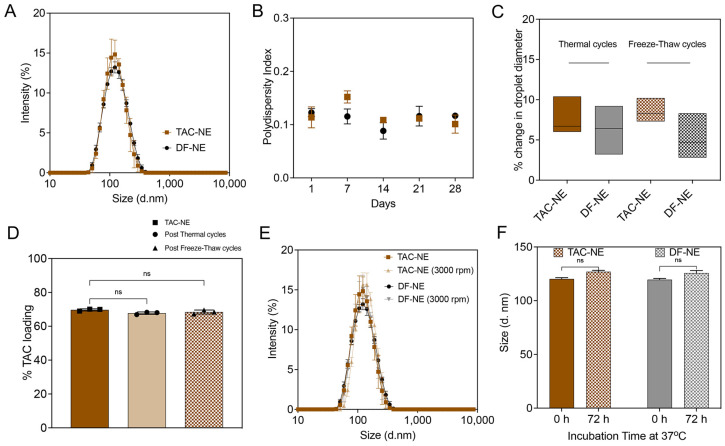
Characterization of Nanoemulsions (TAC-NE and DF-NE). (**A**) Overlay of average size distribution by intensity measured on Day 1. (**B**) One-month follow-up of polydispersity index of NEs. (**C**) Percentage change in droplet diameter after four thermal cycles and freeze-thaw cycles. (**D**) Percentage TAC loading before and after four thermal cycles and freeze-thaw cycles. (**E**) Overlay of the size distribution of NEs before and after centrifugation at 3000 rpm for 30 min. (**F**) Size of NEs stored for 72 h at 37 °C in 10% human serum-containing culture media. Each column represents the mean ± SD (n = 3). ns not significant.

The presented oil-in-water (*o*/*w*) NEs were manufactured on a M110S microfluidizer with certain modifications as compared to our previously reported formulation, where the internal hydrocarbon oil phase was stabilized by a combination of non-ionic surfactants [41]. To increase the loading of a hydrophobic drug (TAC) in the NE droplet, we increased the hydrocarbon oil fraction from the previously reported 8% *w*/*v* to 12.96% *w*/*v* in the new formulation. The hydrocarbon phase consisted of Miglyol 812 (glyceryl tricaprylate/caprate), a medium-chain triglyceride, which is recognized as a GRAS (generally regarded as safe) excipient and was ideal due to its wide applicability to formulate parenteral emulsion [42]. Furthermore, Miglyol 812 has also been reported as a lipid vehicle of choice to formulate TAC nanoemulsion [43]. To emulsify the increased oil fraction in the aqueous phase, non-ionic surfactants CrEL and P105 were used. The TAC-NE and DF-NE were prepared with a total of 5% *w*/*v* surfactant blends, where 2% *w*/*v* was P105 and 3% *w*/*v* was CrEL. Although CrEL (Polyethoxylated castor oil) is a choice solubilizer for TAC [43,44], with a reported solubility of 25.02 ± 1.16 mg/mL [44], it is not a physiologically inert vehicle. Dose-dependent anaphylactoid hypertensive reactions in response to CrEL are documented when used as a solubilizing vehicle in the paclitaxel formulation (Taxol) [45]. However, the paclitaxel formulation consisted of 50% CrEL as a solubilizer, resulting in an administration of 527 mg of CrEL per mL of the formulation. The reported formulation administers 30 mg of CrEL, which is an ~18-fold lower dose than the paclitaxel formulation. As a result, based on the prior reports [41], we do not anticipate severe toxicity; nonetheless, dose-dependent long-term toxicity studies in rodent models and higher primates are warranted in the future. The Hydrophilic-Lipophilic Balance (HLB) value of the CrEL/P105 surfactant blend was calculated as indicated below:

HLB_mix_ = F_CrEL_ HLB_CrEL_ + F_P105_ HLB_P105_,

where HLB_mix_, HLB_CrEL_, HLB_P105_ are the HLB values of mixed surfactants, Kolliphor^®^ EL (CrEL, HLB = 12–14), and Pluronic 105 (P105, HLB = 15), respectively. F_CrEL_, F_P105_ are the weight fractions of CrEL and P105. Further, the developed NEs were characterized for colloidal and fluorescence stability. The size distributions of TAC-NE and DF-NE tightly overlapped each other, with a mean particle size of ~125 nm and a polydispersity index of less than 0.2 (Figure 2A,B). The recorded zeta potential for TAC-NE was −1.58 ± 0.09 mV and DF-NE was −1.57 ± 0.08 mV (Supplementary Figure S1A)

The NEs were exposed to temperature fluctuations, and the percentage change in droplet diameter was measured as an indication of colloidal stability. The recorded percentage change in droplet diameter was less than 10% following four thermal cycles between 50 °C and 4 °C and freeze-thaw cycles between 25 °C and −20 °C (Figure 2C). The percentage TAC loading in the NE was 68.4 ± 1.2%, quantified through RP-HPLC. The exposure of NEs to cyclic temperature fluctuations did not significantly vary TAC loading in the NE droplet (Figure 2D). To model the mechanical and biological stressors, TAC-NE and DF-NE were exposed to high-speed centrifugation for forced separation and to a human serum-containing cell culture medium for 72 h at 37 °C, respectively. No visual phase separation or more than 10% increase in the mean particle diameter were recorded post exposure to the stress conditions (Figure 2E,F).

The NEs were sterilized through a membrane filter with a mean pore diameter of 0.22 µm before incorporation in the NEG. The membrane filtration process did not significantly increase the NE droplet diameter (Supplementary Figure S1B). All the NEs with or without TAC were reproduced at the 27 mL scale on M110S (n = 3), demonstrating a robust manufacturing process.

### 3.2. Preparation of TAC-NEG

Water-soluble triblock linear copolymers of PEO-PPO-PEO were used as thermogelators. The sol-to-gel transition temperature is a function of copolymer concentration. PEO-PPO-PEO copolymer solutions of high concentration are reported to exhibit dramatic changes in viscosity with temperature [35]. Our aim was to select a copolymer concentration that would result in a temperature-sensitive NEG that responds to body temperature and undergoes a temperature-induced sol-gel transition. Al Khateb et al. [33] reported gelation temperatures of binary mixtures of F-68/F-127 in deionized water. Based on this study, the gelation temperature for (F-68/F-127: 2/18% *w*/*v*) was reported to be 38.6 ± 1.8 °C. Another observation from this study was the decrease in gelation temperature with the decrease in the F-68 concentration. As the goal herein was to formulate an injectable NEG that completely gels around physiological temperature, we used 1% *w*/*v* F-68 dissolved in 1XPBS and 18% *w*/*v* of F-127 in water, with a total pluronic concentration of 19% *w*/*v*.

**Figure 3:**
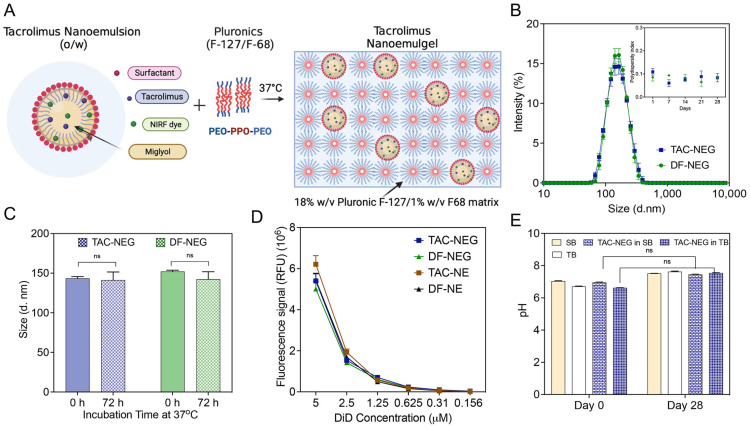
Characterization of Nanoemulgels (TAC-NEG and DF-NEG). (**A**) Illustration of TAC-NE incorporated into the mixed pluronics (Pluronic F127/F68). (**B**) Overlay of average size distribution by intensity and one-month follow-up of polydispersity index. (**C**) Size of NEs stored for 72 h at 37 °C in 10% human-serum containing cell culture media. (**D**) NIRF signal comparison performed at the same settings between NEGs and NEs. (**E**) Sterility testing of TAC-NEG by pH measurements in Soy broth (SB) and Thioglycolate broth (TB) at room temperature. Each column represents the mean ± SD (n = 3). ns not significant.

The colloidally and fluorescently stable sterile filtered TAC-NE and DF-NE were incorporated in the copolymer solution to form TAC-NEG and DF-NEG, respectively (Figure 3A). The mean particle diameter of NEs post release from NEGs was ~150 nm, with a polydispersity index of <0.3 over a month (Figure 3B). As compared to the NEs (Figure 2A), approximately a 25-nm increase in droplet diameter was observed post incorporation and release from the NEG. The zeta potential of TAC-NEG was −1.69 ± 0.28 mV and DF-NEG was −1.17 ± 0.27 mV (Supplementary Figure S1A). Forced separation of NEG by high-speed centrifugation did not result in separation of NE from the gelling components visually inspected along with no change in the NE droplet diameter (Supplementary Figure S1C). Similarly, we did not observe more than 10% change in the droplet diameter of the released NE when NEGs were dissolved in high-FBS-containing cell culture media and incubated at 37 °C for 72 h (Figure 3C). The fluorescent signal intensities for TAC-loaded and DF-NE and NEG tightly overlapped and showed a linear correlation with the DiD-labeled NE concentration, which is important for reliable comparison of macrophage uptake (Figure 3D). Further, the pH of TAC NEG was 7.14, which is suitable for injectable applications (Supplementary Figure S1D).

Thermosensitive NEGs cannot withstand common end-stage sterilization techniques, such as autoclaving, radiation, or chemical sterilization, as it can result in the destabilization of NE droplets and loss of polymer gelling properties. Although an aseptic production process was utilized, this work confirms the sterility of the finished NEG product by visually inspecting for microbial growth and recording pH changes. We did not observe any changes in the pH due to the microbial growth or cloudiness in the growth media (fluid thioglycolate broth and Trypcase soy broth), confirming the sterility of the final product (Figure 3E). This suggests there was no loss of sterility during the planetary mixing of the components. Negative controls consisting of each of the microbial growth media that had not been intentionally contaminated were included in the assay, for confirming the sterility of the used reagents. The percentage of TAC incorporated in NEG was around 100% as the manufacturing process did not involve multiple transfers or steps leading to TAC degradation that would contribute to any drug loss. The final concentration of TAC in TAC-NEG was approximately 318 µM.

### 3.3. Release of TAC-NE from TAC-NEG

**Figure 4:**
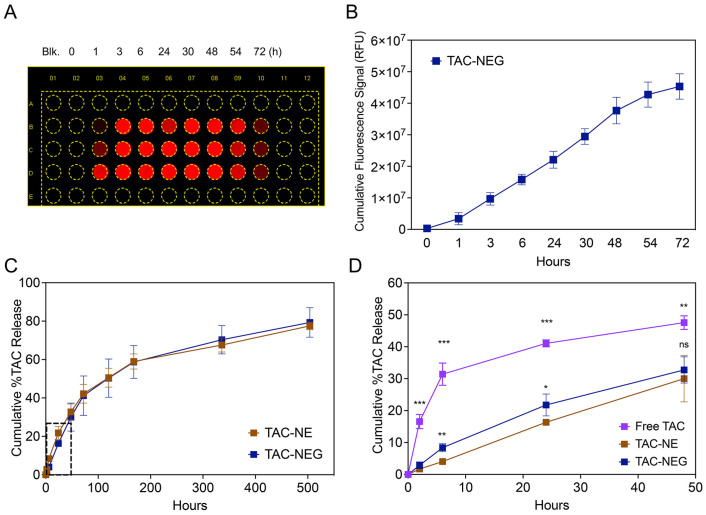
Release of NE from NEG and In vitro release data. (**A**) Representative image of a multiwell plate exposed to 700 nm channel (DiD) on a LICOR Odyssey imager. (**B**) NIRF signal (RFU) from the NE droplets released from TAC-NE, as it is dissolved over a period of 72 h. (**C**) Comparison of in vitro release between TAC-NE and TAC-NEG in 1X PBS with 0.1% Tween 80 (pH = 7.4) followed for 21 days. (**D**) Comparison of in vitro release between TAC-NE, TAC NEG, and TAC solution (pH = 7.4) for the first 48 h (expansion on the dotted box in Figure 3C). The data are expressed as mean values ± SD (n = 3). *** *p* < 0.0005, ** *p* < 0.005, * *p* < 0.05, ns not significant.

To demonstrate the ability of NIRF-labeled NE to release from gelled NEG, we quantified the fluorescence signal generated from the released NE droplets in the FBS-containing cell culture medium kept at physiological temperature (Figure 4A). We observed a time-dependent increase in the fluorescent signal as the NEG dissolved, releasing the NE droplets. The pluronic-based NEG completely dissolved in 72 h when tested at a 1:5 ratio between the gelled NEG and release media (Figure 4B).

The in vitro release of TAC from TAC-NE, TAC-NEG, and free TAC was compared at 37 °C ± 0.5 °C (Figure 4C,D). The percentage cumulative release of TAC from NEG was significantly lower in the initial timepoints (6 h and 24 h) as compared to NE (Figure 3D). However, as the NEG dissolved, the release of TAC from NE and NEG matched in the later timepoints (Figure 4C). Further, as compared to the TAC solution (16.55 ± 2.23%), the release of TAC was significantly lower from both NE (2.87 ± 1.08%) and NEG (1.72 ± 0.27%) within 2 h (Figure 4D). The release pattern from TAC-NEG was more sustained, as at the end of the study (Day 21) we observed a 79.31 ± 2.00% cumulative release of TAC.

### 3.4. Rheology

**Figure 5:**
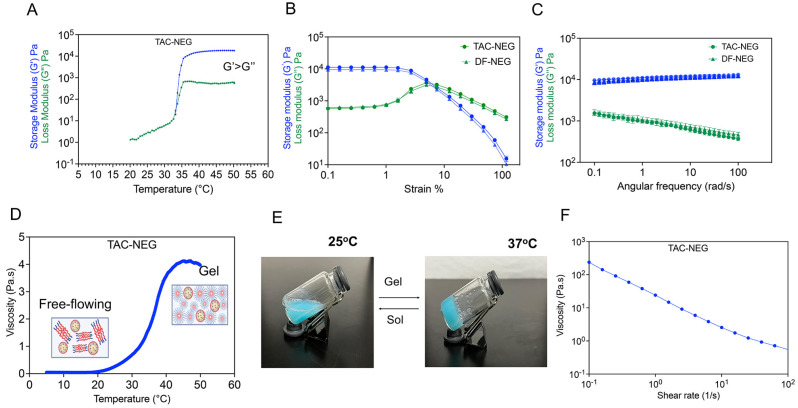
Rheological behavior of the Control gel, TAC-NEG, and DF-NEG. (**A**) Plot of storage (G’) and loss (G”) moduli as a function of temperature (°C) for TAC-nanoemulgel. (**B**,**C**) Oscillation Amplitude (OA) and Oscillation Frequency (OF) sweeps on TAC-NEG and DF-NEG. OA tests were performed using 10 rad/s at strains of 0.1% to 100%. OF tests were performed with a constant strain of 0.1% and varying angular frequency from 0.1 rad/s to 100 rad/s on TAC-NEG and DF-NEG. (**D**) Viscosity change of TAC-NEG in response to temperature increase from 5 °C to 50 °C using constant shear of 100 1/s. (**E**) A photograph of TAC-NEG exposed to 37 °C for 5 min. (**F**) Plot of viscosity verses shear rate at ambient temperature.

The thermogelling and viscoelastic behavior of TAC-NEG, DF-NEG, and the control gel were studied based on rheological measurements. The storage (G’) and loss modulus (G”) were plotted as a function of temperature, starting from 20 °C to 50 °C. We observed that, around 35 °C, both the moduli had a crossover, indicating the gel point for TAC-NEG. The gel point for DF-NEG was observed around 36 °C, indicating no effect of the drug. Both the DF-NEG and TAC-NEG completely form gel at physiological temperature, as the storage modulus was one order of magnitude higher than the loss modulus (Figure 5A). Based on the oscillation amplitude sweep data at constant angular frequency, the storage and loss moduli were independent of the applied oscillation strain, below a strain amplitude of 2%. This was the linear viscoelastic region (Figure 5B). The presence of TAC in the NE droplet did not alter the gel strength. On increasing the applied strain further, both the moduli deviated from linearity, and G’ sharply decreased, whereas G” reached a maximum before undergoing a gradual decay. The oscillation frequency sweep data showed that G’ remains relatively independent of the angular frequency and the power exponent of the G” becomes negative, indicating non-Newtonian viscoelastic behavior of the NEGs. Furthermore, the resultant phase angle (tan δ) < 1 at all frequencies. The nanoemulgel behaves more like a viscoelastic solid than a viscous liquid at physiological temperature (Figure 5C). This behavior is important for their application as injectable gels. The temperature ramp study showed that as the phase transition temperature increased, there was a consequent increase in the viscosity of the NEGs (Figure 5D). The NEGs quickly responded to the temperature changes, which is critical as these NEGs are designed to form drug delivery depots when applied to the graft at physiological temperature. The sol-to-gel transition was visually confirmed by performing a vial inversion test at 37 °C (Figure 5E). At ambient temperature, we observed a decrease in viscosity with an increase in shear rate, demonstrating the shear-thinning behavior desired for injectable NEG formulations (Figure 5F). The same set of rheological measurements were performed on the control gel without NE (Supplementary Figure S3A–C).

### 3.5. Macrophage Viability and Cellular Uptake of TAC

**Figure 6:**
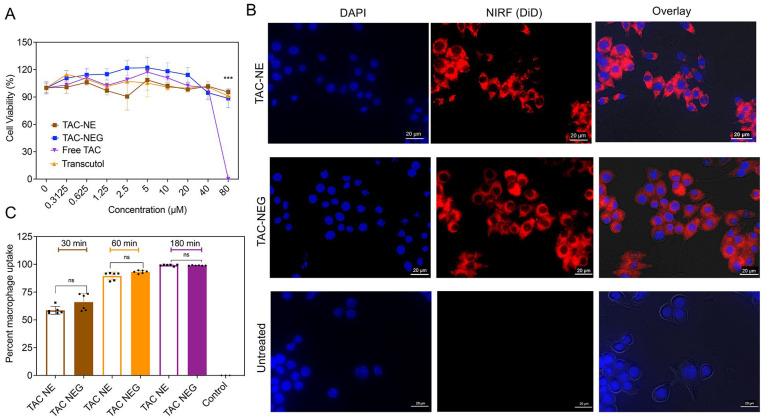
Macrophage viability and cellular uptake. (**A**) Macrophage viability was determined via ATP-based CellTiter-Glo^®^ 2.0 assay post exposure to TAC-NE, TAC-NEG, free TAC, and free drug vehicle: Transcutol for 24 h. The data represents mean ± SD (n = 6), *** *p* < 0.0005. (**B**) Representative images of RAW 264.7 macrophages exposed to concentration-matched DiD-labeled TAC-NE and TAC-NEG for 6 h and imaged for DAPI nuclei staining, Cy5 cytoplasm staining, and overlay of brightfield, DAPI, and Cy5. (**C**) Comparison of cellular uptake of TAC-NE and TAC-NEG in LPS-activated macrophages determined by flowcytometry analysis. The data are shown as the mean ± SD (n = 6/group independent cell culture), and 40,000 cells were counted. ns not significant.

An ATP-based CellTiter-Glo^®^ luminescent assay was used to study the viability of RAW 264.7 macrophages exposed to the NEs and NEGs with or without TAC for 24 h. We did not observe a decrease in ATP levels due to varying concentrations of NEs or NEGs as compared to the untreated cells. This reflected macrophage viability in the presence of NEs and NEGs even at higher concentrations (80 μM). However, TAC dissolved in transcutol (TAC solution) at 80 μM resulted in significant toxicity in RAW 264.7 macrophage which was due to the drug alone, as volume-matched transcutol did not demonstrate any cytotoxic effects (Figure 6A). The effect of NEs and NEGs was further evaluated on lipopolysaccharide (LPS)-activated macrophages (Supplementary Figure S4).

The binding and internalization of TAC-NE and TAC-NEG in macrophages were qualitatively tracked by using microscopy (Figure 6B). The DiD-labeled NEs and NEGs were taken up by macrophages and appeared to be distributed in the cytoplasm around the nucleus as punctate patterns. Furthermore, we performed quantitative uptake studies on LPS-activated RAW 264.7 macrophages by performing flow cytometry. By comparing the uptake of TAC-NE to that of TAC-NEG added at the same DiD concentration, we observed a time-dependent macrophage uptake. The macrophage uptake was not significantly different between concentration-matched TAC-NE and TAC-NEG at the exposure time. After 180 min of exposure, the uptake reached saturation, showing ~98% of NE and NEG uptake by activated macrophages (Figure 6C).

### 3.6. Enzyme-Linked Immunosorbent Assay

**Figure 7:**
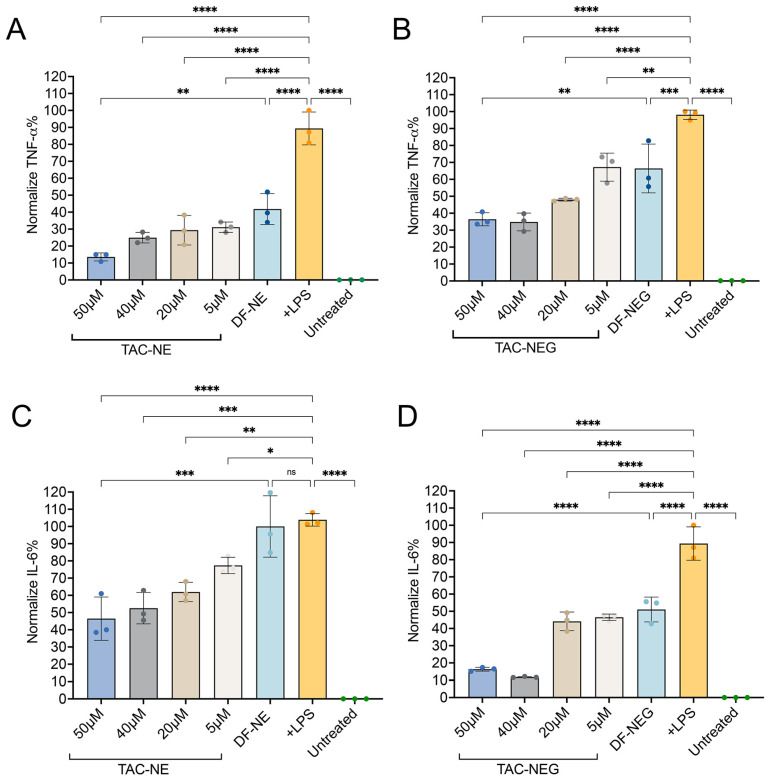
TAC-NE, DF-NE, TAC-NEG, DF-NEG suppress pro-inflammatory cytokine release from activated RAW 264.7 macrophages. (**A**) Dose-dependent inhibition of TNF-α release from LPS-activated macrophages exposed to TAC-NE and DF-NE. (**B**) Dose-dependent suppression of TNF-α release from LPS-activated macrophages exposed to TAC-NEG and DF-NEG. (**C**) Dose-dependent suppression of IL-6 release from LPS-activated macrophages exposed to TAC-NE and DF-NE. (**D**) Dose-dependent suppression of IL-6 release from LPS-activated macrophages exposed to TAC-NEG and DF-NEG. Each bar represents mean ± SD (n = 3, independent culture) **** *p* < 0.00005, *** *p* < 0.0005, ** *p* < 0.005, * *p* < 0.05, ns not significant.

Next, we investigated the anti-inflammatory effects of TAC-NE and TAC-NEG to decrease the release of two cytokines (IL-6 and TNF-α) from LPS-activated RAW 264.7 macrophages using an ELISA assay. The results showed that the levels of cytokines released from LPS-activated macrophages were significantly higher than those of the untreated group (without LPS stimulation) (Figure 7A–D). Both TAC-NE and TAC-NEG treatments resulted in a concentration-dependent decrease in TNF-α release. In the case of TAC-NE, the highest concentration tested (50 μM) resulted in an 85% decrease in TNF-α release, while the lowest tested concentration (5 μM) led to a 65% decrease (Figure 7A). Similarly, treatment with TAC-NEG also exhibited a dose-dependent reduction in TNF-α release (Figure 7B), with the highest concentration causing a 63% decrease. Interestingly, volume-matched DF-NE and NEG to the highest concentration did decrease the TNF-α release. However, there was a statistically significant difference in TNF-α suppression between TAC-loaded NE and NEG and DF-NE and NEG. This difference suggests that the observed effects were due to the release of TAC from the NE and NEG systems.

We further investigated the effect of TAC-NE (Figure 7C) and TAC-NEG (Figure 7D) on IL-6 release from LPS-activated macrophages. Both the TAC-loaded NE and NEG resulted in a dose-dependent decrease in IL-6. The results showed that treatment with the highest tested concentration of TAC-NEG resulted in an approximately 82% decrease in IL-6 following LPS exposure. Although the volume-matched DF-NEG did show a decrease in IL-6, there was a significant difference in the suppression of IL-6 between TAC-loaded and DF-NEGs.

### 3.7. Nitric Oxide Assay

**Figure 8:**
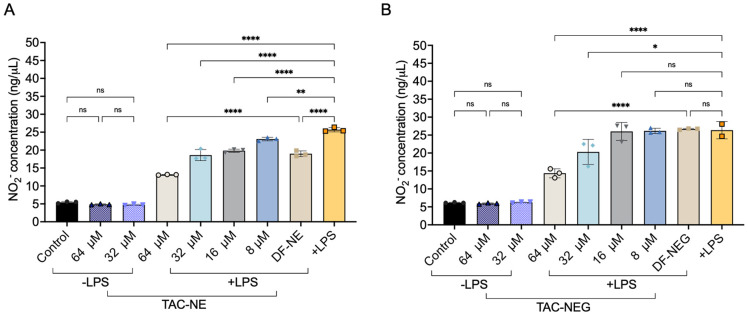
Nitric oxide inhibition (**A**) NO inhibition quantified by measuring the decrease in NO_2_^_^ from LPS-activated macrophages treated with TAC-NE (8–64 μM) and volume-matched to highest concentration DF-NE. (**B**) NO_2_^_^ inhibition from LPS-activated macrophages treated with TAC-NEG (8–64 μM) and volume-matched to highest concentration DF-NEG. Each bar represents mean ± SD (n = 3, independent culture) **** *p* < 0.00005, ** *p* < 0.005, * *p* < 0.05, ns not significant.

In response to LPS stimulation, RAW 264.7 macrophages significantly produced nitrite (NO_2_^−^), an oxidative product of NO, as compared to the control group (absence of LPS) (Figure 8A,B).

We observed a dose-dependent (8–64 μM) decrease in NO_2_^−^ production in the presence of TAC-NE and TAC-NEG. Particularly, activated macrophages treated with 64 μM and 32 μM of TAC-NEG resulted in approximately 39% and 15% decreases in NO_2_^−^ production as compared to positive LPS, respectively. However, when treating with lower concentrations (8 μM and 16 μM) of TAC-NEG, we did not observe a statistically significant decrease, although TAC-NE was able to produce a ~10% decrease in NO_2_^−^ production when treated at these concentrations. Additionally, treating RAW 264.7 macrophages with the highest concentrations (64 μM and 32 μM) of TAC-NE and TAC-NEG in the absence of LPS did not increase NO_2_^−^ production. Overall, both nanosystems were able to deliver TAC to activated macrophages and decrease nitric oxide.

### 3.8. Long-Term Stability Studies

**Figure 9:**
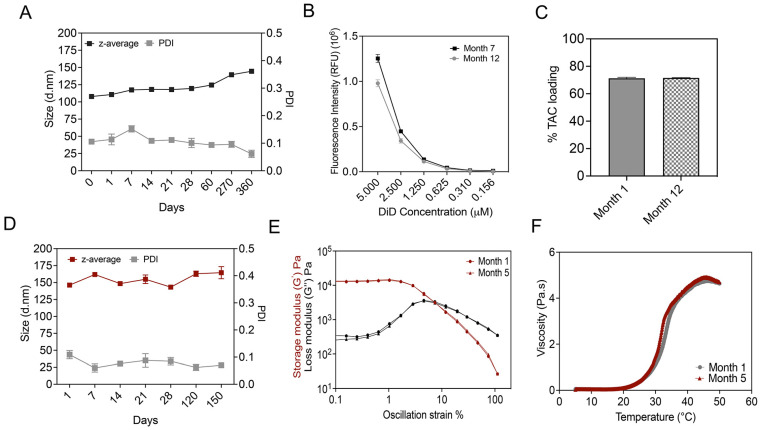
Long-term stability studies. (**A**) Size and polydispersity follow-ups performed over a year on TAC-NE. (**B**) Comparison of fluorescence intensity measured during Month 7 and Month 12 follow-up. (**C**) Comparison of tacrolimus loading in NE measured at Month 1 versus Month 12. (**D**) Size and polydispersity follow-ups performed over five months on TAC-NEG. (**E**) Comparison between OA sweep on TAC-NEG measured at Month 1 and Month 5. OA tests were performed using 10 rad/s at strains of 0.1% to 100%. (**F**) Comparison of change in viscosity with temperature for TAC-NEG over a period of five months.

We evaluated the long-term stability of TAC-NE by measuring changes in the droplet diameter, PDI, DiD fluorescence signal intensity, and drug loading over a period of ten months (Figure 9A–C). We observed a gradual increase in droplet diameter as a function of time. The droplet diameter increased by 30 nm at the end of a year follow-up; however, the PDI remained <0.2 during the same period. Although the change in droplet diameter with time was anticipated, as NEs are kinetically stable systems, the size of NEs remained within the specifications for successful macrophage uptake (droplet diameter < 200 nm). Over a period of twelve months, the percentage of TAC loaded in NE did not significantly change, indicating an absence of drug precipitation or NE droplet destabilization (Figure 9C). Furthermore, we also performed a stability assessment on TAC-NEGs over a period of five months by determining changes in droplet diameter, PDI, and rheological properties (Figure 9D–F). TAC-NEG remained stable as we did not observe changes in size, PDI, or rheological properties. During the assessment period, all the formulations were stored at 4 °C.

We further showed manufacturing consistency by reproducing batches of tacrolimus-loaded DF-NEs and -NEGs at different time intervals by different operators. The minimal variation in the size distribution overlays suggests the manufacturing processes’ reproducibility (Supplementary Figure S5A–D). This will further help in conducting reliable in vivo studies, as any significant variations in the product could introduce confounding factors, ultimately affecting the accuracy of the obtained data.

## 4. Discussion

In this work, we developed a formulation that can intracellularly deliver both a drug (TAC) and NIRF dye (DiD) payload to activated macrophages, which are known drivers of organ rejection and poor outcomes following organ transplantation. Our theranostic platform is thus suitable for site-specific delivery into grafts and could help track macrophage dynamics during AR or CR in transplants. Such local delivery of TAC can enable increased graft tissue concentrations of the drug and site-specific, regional immunomodulation with reduced or negligible systemic exposure [27,28,29].

The colloidally and fluorescently stable NE was formulated with a high hydrocarbon oil fraction to incorporate a targeted amount of TAC and a lipophilic NIRF dye. At a specific surfactant concentration, increasing the oil fraction (dispersed phase) can increase droplet diameter by increasing the collision rate between the droplets [46]. However, on increasing the percentage of Miglyol 812 from 8% *w*/*v* to 12.96% *w*/*v* at a fixed surfactant concentration, we did not observe significant changes in the droplet diameter. Sarheed et al. reported a similar observation, where at a specific surfactant concentration, changing the oil fraction of beeswax and coconut oil did not significantly change the droplet diameter [46]. On the contrary, increasing the percentage of oleic acid significantly increased the droplet diameter. Thus, this behavior depends on the type of oil and surfactant-to-oil ratio used in the formulation. Monitoring the stability of NEs under different stress conditions is important to ensure their performance throughout their shelf life. Physical stress tests such as centrifugation and filtration can accelerate the rate of droplet coalescence [47]. Exposing NEs to cyclic high-low temperatures increases the rate of Ostwald ripening and changes the interfacial tension [48], whereas freeze-thaw cycles can result in oil crystallization and conformational changes in the interfacial layer [49]. As a result, these physical stressors can result in NE droplet destabilization, which can manifest as a change in droplet diameter or phase separation. The TAC-loaded and DF-NEs were stable, as we did not observe more than a 10% change in the droplet diameter or phase separation after any physical stress tests. Furthermore, the formulations were suitable for in vitro and future in vivo work as the droplet diameter was maintained in the presence of serum proteins, salts, or nutrients.

We used pluronic copolymers (F-127/F-68: 18/1 *w*/*v*), which have been well studied for their “thermoreversible gelation” when used in low as well as high concentrations [33,36,50]. Along with Pluronic F-127, Pluronic F-68 was introduced as an additive as it reduces gel dissolution and provides sustained release [51]. Additionally, F-68 reduces viscosity and increases the gelation temperature, which is important for injectability at the graft site [33]. A previous study has shown that the presence of the oil phase (or the NE) is critical for observing thermogelling behavior when working with low F-127/F-68 concentrations [36]. We do not observe this dependency in this work with a higher concentration of pluronics, as the sol-to-gel transition was observed in the control gel without NE. The gelation mechanism for the pluronic-based gel is due to the creation of a 3D cubic lattice of arranged spherical micelles at high temperatures, overall increasing the viscosity [52,53]. The z-average size of the NE released out of the NEG increased by ~25 nm, but the PDI remained <0.1 with no changes in zeta potential. Indeed, the droplet diameter was within specifications for efficient macrophage uptake. Since the platform was designed for direct application to the graft, we determined the sterility of NEG post-manufacturing. This is important from a biomedical standpoint for minimizing the risk of graft failure due to infections. Despite the limited literature on the microbiological testing of thermosensitive NEGs, the microbiological tests supported the conclusion that aseptic planetary mixing can be successfully adopted to manufacture sterile NEGs. We also demonstrated the release of NE droplets from the NEG system by quantifying the NIRF signal at regular intervals. The F-127/F-68 gel completely dissolved in approximately 24 h in the release media when the ratio between the gel and release media was 1:5. Based on the in vitro drug release data, at the initial time points (up to 24 h) the release of TAC from NEG was significantly lower as compared to NE. However, after 24 h, the TAC released from both systems equalizes, overall showing a sustained drug release profile. We predict this could be due to the complete dissolution of TAC-NEG in the presence of excess release media in the dialysis bag by the end of 24 h. Importantly, our pluronic-based NEG did not show initial burst release as compared to the TAC solution, as well as previously reported TAC solid lipid nanoparticles and TAC transferosomes incorporated into Carbopol-based gels [54,55]. The formulation behaves as a viscous liquid (sol) at low temperatures (<20 °C) and forms a stable gel at physiological temperature, which will allow it to act as a local depot capable of sustained release. The NEs were easily injectable through a 21 G syringe and were able to quickly gel in response to temperature changes near body temperature (37 °C).

For in vitro studies, we first showed that all the manufactured formulations were non-toxic to RAW 264.7 mouse macrophages in the tested concentration range. The uptake of TAC-NE and TAC-NEG by activated macrophages was time-dependent, almost reaching saturation within 180 min of treatment exposure. As expected, we did not observe significant differences in activated macrophage uptake rates when comparing NE droplets released from NEG and NE alone. Increased levels of macrophage-associated pro-inflammatory cytokines such as TNF-α and IL-6 are strongly associated with primary graft dysfunction, neuroinflammation, and an increased probability of organ rejection [56,57,58,59]. The increased levels of TNF-α and IL-6 are also reported to augment the expression of macrophage-associated allograft inflammatory factor-1 (AIF-1, a cytokine-responsive inflammatory protein), which increases the risk of rejection [60,61]. In this study, we showed a dose-dependent decrease in TNF-α and IL-6 secretions with both TAC-loaded NE and NEG treatments. Moreover, macrophage activation increases the levels of nitric oxide synthase 2, consequently triggering high NO output and contributing to cell toxicity in the graft [62]. We observed that treatments with TAC-loaded NE and NEG resulted in a concentration-dependent decrease in NO_2_^−^ concentration, a main oxidative product of NO. This was consistent with a previous study in LPS-activated rat macrophages showing a decrease in NO levels in the presence of TAC [63]. Lastly, monitoring the long-term stability of NEs and NEGs was critical, as it can be compromised depending on the storage conditions. All the formulations were suitable for future in vivo work and scale-up applications, as we did not observe any significant time-dependent instability in NEs and NEGs when stored at 4 °C during periods of twelve and five months, respectively.

## 5. Conclusions and Translational Relevance

Taken together, we formulated a reversibly thermoresponsive NEG with theranostic capability that offers promise in local graft tissue and macrophage-targeted delivery of TAC-loaded nanoemulsions at the site of organ rejection. This preliminary, in vitro validation study confirms that TAC-NEGs are non-toxic and taken up by activated macrophages. Treatment with TAC-NEG significantly reduced proinflammatory cytokines such as TNF-α, IL-6, and the proinflammatory mediator nitric oxide in macrophage cultures. The NEs and NEGs showed remarkable physical stability and serum resistance. The NEGs showed a sustained release pattern, avoiding burst exposure of TAC to the macrophages. This approach opens a wide range of avenues for exploring innovative biomedical and pharmaceutical applications of NEGs in transplantation. In contrast to the prevailing standard-of-care approach of non-specific immunosuppression of the recipient, this TAC-loaded NIRF-NEG platform offers an injectable, theranostic platform that allows graft-embedded drug delivery of ultra-low doses of TAC and/or other drugs/payloads with unique cell/molecular targets of action against both AR and, most importantly, CR. Macrophage location and activity can be non-invasively monitored with NIRF dye-labeled NEs. This inbuilt theranostic capability of the proposed platform helps monitor TAC release from NEG in the presence of macrophages and assess drug efficacy and exhaustion. Further evaluation of in vivo transplant models as standalone or as adjunctive treatment will be the focus of our future studies.

## Data Availability

Data can be made available for non-commercial uses and per reasonable request made to the corresponding author at janjicj@duq.edu.

## Supplementary Materials

The following supporting information can be downloaded at: https://www.mdpi.com/article/10.3390/pharmaceutics15102372/s1, Figure S1. Characterization of NE and NEGs with or without TAC. Figure S2. HPLC calibration curve and representative of HPLC chromatogram. Figure S3. Rheological measurements on F-127/F-68 control gel. Figure S4. LPS-activated macrophage viability in presence of TAC-NE, TAC-NEG, DF-NE, and DF-NEG. Figure S5. Overlays of size distribution for batch reproducibility. Figure S6. Gating information for Flowcytometry.
