# Supplementary material for: A Reversibly Thermoresponsive, Theranostic Nanoemulgel for Tacrolimus Delivery to Activated Macrophages: Formulation and In Vitro Validation
Source: Pharmaceutics. 2023 Sep 22;15(10):2372. doi: 10.3390/pharmaceutics15102372 (PMC10610217; doi:10.3390/pharmaceutics15102372)
Supplement: Supplementary file 1 [file pharmaceutics-15-02372-s001.zip › pharmaceutics-2612504-supplementary.pdf]

A Reversibly Thermoresponsive, Theranostic Nanoemulgel for Tacrolimus Delivery to Activated Macrophages: Formulation and In Vitro Validation

Riddhi Vichare [1], Caitlin Crelli [1], Lu Liu [1], Amit Chandra Das [1], Rebecca McCallin [1], Fatih Zor [2], Yalcin Kulahci [2], Vijay S. Gorantla [2] and Jelena M. Janjic [1,*]

[1] School of Pharmacy, Graduate School of Pharmaceutical Sciences, Duquesne University, Pittsburgh, PA 15282, USA; vicharer@duq.edu (R.V.); crellic@duq.edu (C.C.); liul@duq.edu (L.L.); dasa@duq.edu (A.C.D.); mccallinr@duq.edu (R.M.)

[2] Wake Forest School of Medicine, Wake Forest Institute of Regenerative Medicine, Winston Salem, NC 27101, USA; fzor@wakehealth.edu (F.Z.); ykulahci@wakehealth.edu (Y.K.); vgorantl@wakehealth.edu (V.S.G.)

* Correspondence: janjicj@duq.edu

SUPPLEMENTAL FIGURES AND LEGENDS

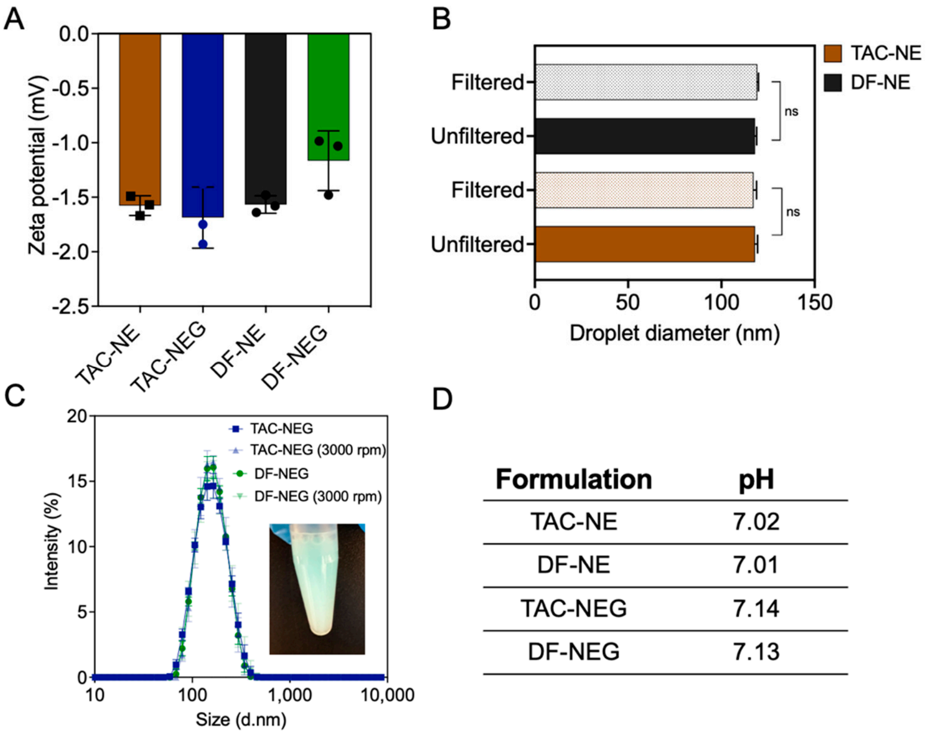

Formulation	pH
TAC-NE	7.02
DF-NE	7.01
TAC-NEG	7.14
DF-NEG	7.13

Supplementary Figure S1. (A) Zeta potential (mV) measurements for TAC-NE, DF-NE, TAC-NEG, and DF-NEG measured on Day 1. (B) Size of TAC loaded and DF NEs before and after filtration through a membrane filter with a mean pore diameter of 0.22 µm. (C) Overlay of the size distribution of TAC loaded and DF NEs before and after centrifugation at 3000 rpm for 30 mins. (D) Average of recorded pH

measurements for TAC-NE, DF-NE, TAC-NEG, and DF-NEG. Each data is represented as the mean ± SD (n=3). ns not significant.

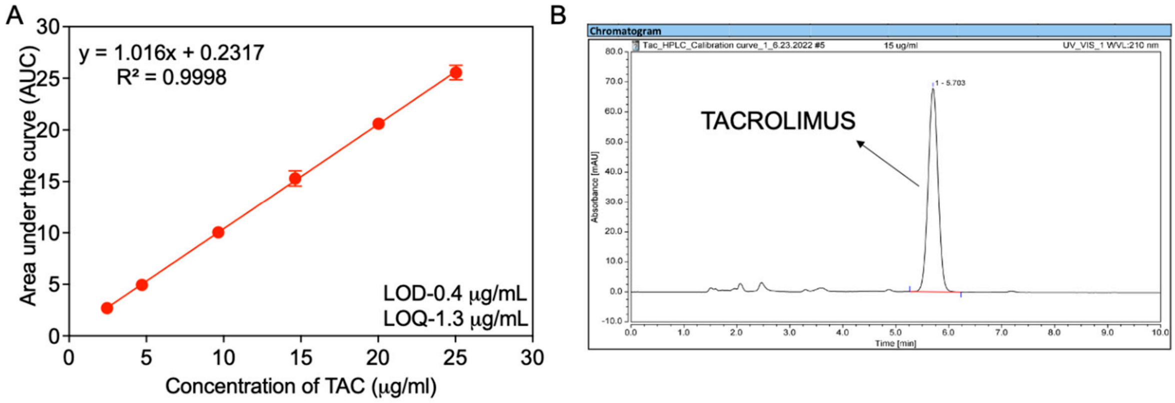

Supplemental Figure S2: (A) HPLC method validation standard curve for Tacrolimus with limit of detection (LOD) and limit of quantification (LOQ). (B) Representative of HPLC chromatograph indicating Tacrolimus peak using ACN: Water: Orthophosphoric acid (75:25:0.1) as the mobile phase on a C18 column with UV detection at 240 nm. The retention time for TAC was 5.8 min.

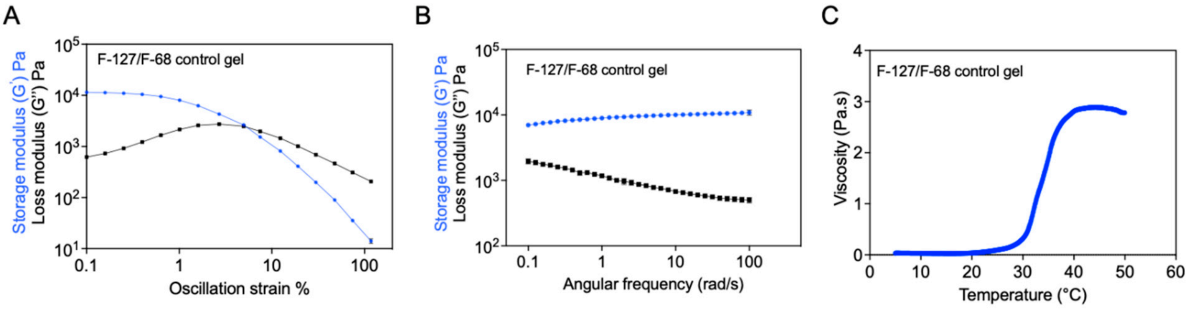

Supplementary Figure S3. (A,B) Oscillation Amplitude and Oscillation Frequency sweeps on F-127/F-68 control gel. OA tests were performed using 10 rad/s at strain of 0.1 to 100%. OF tests were performed with a constant strain of 0.1% and varying angular frequency from 0.1 rad/s to 100 rad/s on F-127/F-68 control gel. (C) Viscosity change of F-127/F-68 control gel in response to temperature increase from 5°C to 50°C using constant shear of 100 1/s.

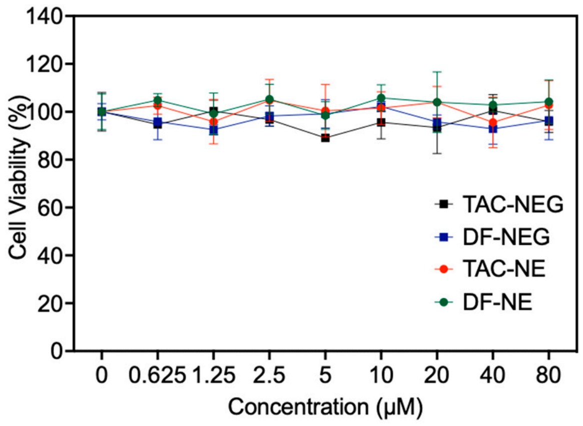

Supplementary Figure S4. LPS-activated macrophages were exposed to TAC-NE, TAC-NEG, DF-NE, and DF-NEG. Macrophage viability assessed via ATP-based CellTiter-Glo® 2.0. The data points represent mean ± SD (n= 6).

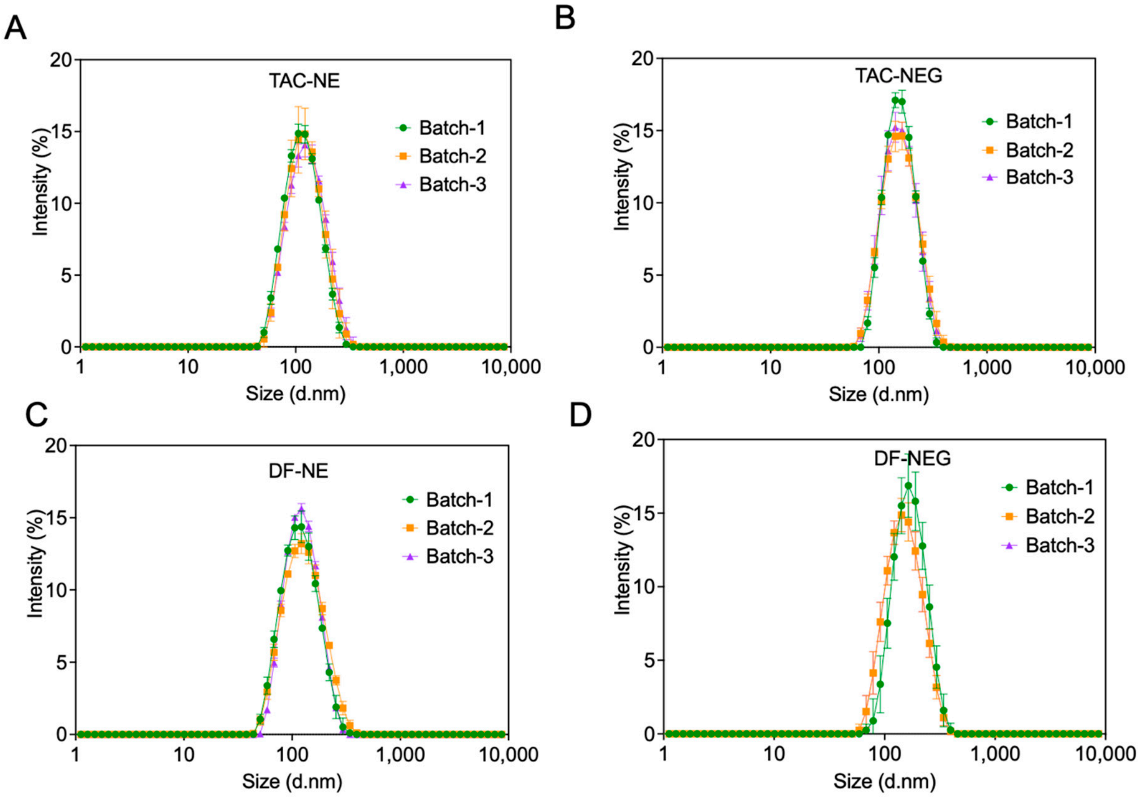

Supplementary Figure S5: (A-D) Overlays of averaged size distributions from three reproducible batches of TAC-NE, TAC-NEG, DF-NE, and DF-NEG produced on M110S (25 mL).

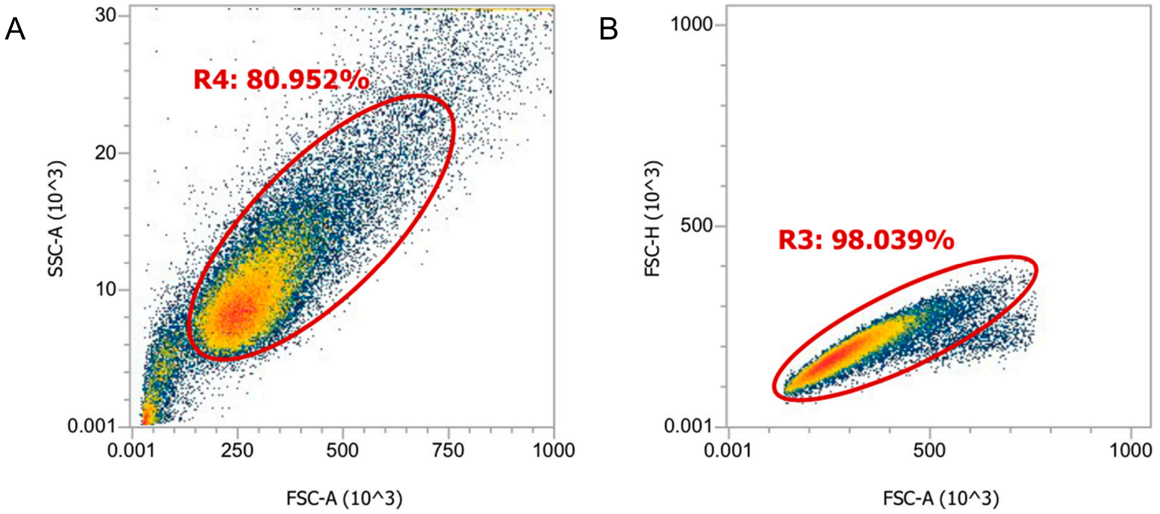

Supplementary Figure S6: (A, B) All the gating was set on the target population (FSC-A vs SSC-A) followed by gating singlet gating. DiD was detected in RL1 channel of Attune Nxt flow cytometer.
